# Facile Synthesis of *N*-(4-Bromo-3-methylphenyl)pyrazine-2-carboxamide Derivatives, Their Antibacterial Activities against Clinically Isolated XDR *S. Typhi*, Alkaline Phosphatase Inhibitor Activities, and Docking Studies

**DOI:** 10.3390/ph17091241

**Published:** 2024-09-20

**Authors:** Abdul Hannan Khan, Muhammad Bilal, Abid Mahmood, Nasir Rasool, Muhammad Usman Qamar, Muhammad Imran, Sebastian Ionut Toma, Oana Andreescu

**Affiliations:** 1 Department of Chemistry, Government College University Faisalabad, Faisalabad 38000, Pakistan; hannankhan786173@gmail.com (A.H.K.); muhammadbilalgcuf@gmail.com (M.B.); 2 Department of Pharmaceutical Chemistry, Government College University Faisalabad, Faisalabad 38000, Pakistan; abid9550@yahoo.com; 3 Institute of Microbiology, Faculty of Life Sciences, Government College University Faisalabad, Faisalabad 38000, Pakistan; musmanqamar@gcuf.edu.pk; 4 Chemistry Department, Faculty of Science, King Khalid University, P.O. Box 9004, Abha 61413, Saudi Arabia; imranchemist@gmail.com; 5 Faculty of Medicine, Transilvania University of Brasov, 500036 Brasov, Romania; sebitom2002@yahoo.com (S.I.T.); andreescu_o@yahoo.com (O.A.)

**Keywords:** pyrazine-2-carboxamides, XDR *S. Typhi*, alkaline phosphatase inhibitors, drug resistance, docking studies

## Abstract

The emergence of extensively drug-resistant *Salmonella* Typhi (XDR-*S. Typhi*) poses a grave public health threat due to its resistance to fluoroquinolones and third-generation cephalosporins. This resistance significantly complicates treatment options, underscoring the urgent need for new therapeutic strategies. In this study, we synthesized pyrazine carboxamides (**3, 5a**–**5d**) in good yields through the Suzuki reaction. Afterward, we evaluate their antibacterial activities against XDR-*S. Typhi* via the agar well diffusion method; **5d** has the strongest antibacterial activity with MIC 6.25 (mg/mL). Moreover, in vitro Alkaline Phosphatase inhibitor activity was also determined; **5d** is the most potent compound, with an IC_50_ of 1.469 ± 0.02 µM. Further, in silico studies were performed to find the type of interactions between synthesized compounds and target proteins.

## 1. Introduction

The Gram-negative bacterium, *Salmonella* enterica subspecies enterica serovar Typhi (*S. Typhi*), is a causative agent of typhoid fever transmitted through contaminated water and food [1]. The disease is prevalent primarily in low-income communities (LMICs) with fragile health infrastructure and improper sewage systems [2]. Worldwide, there are thought to be 26.9 million cases of typhoid fever per year (interquartile range: 18.3–35.7 million) [3]. Although there is significant variation in the distribution of typhoid fever, it is believed that the Indian and African subcontinents have the highest disease burden [4]. The term multidrug-resistant *S. Typhi* (MDR-*S. Typhi*) has been used to describe resistance to ampicillin, co-trimoxazole, chloramphenicol, and fluoroquinolones [5]. These pathogens acquired resistance due to the acquisition of antimicrobial resistance gene (ARG) [6] cassettes located on self-transmissible plasmids or integrated into chromosomes, which is more common than previously thought [7]. However, antibiotic selection maintains ARGs on plasmids [8]. The World Health Organization (WHO) ranked XDR-*S. Typhi* in the “High Priority Pathogens List”. XDR-*S. Typhi* was first reported in Hyderabad, Sindh, Pakistan, in 2016 [9]. These pathogens cause clinical severe infections, including bacteremia and septicemia, and produce resistance to ampicillin, ciprofloxacin, cotrimoxazole, and ceftriaxone, and are only sensitive to azithromycin and carbapenems [10].

Consequently, innovative therapeutic strategies are required to address the AMR issue. The debate about the long-term efficacy of antimicrobial drugs continues since it is becoming more challenging to treat developing multidrug-resistant infections with present therapies [11]. Developing novel antimicrobial drugs and implementing a comprehensive plan is crucial in the fight against the emergence of microbial resistance [12].

**Figure 1:**
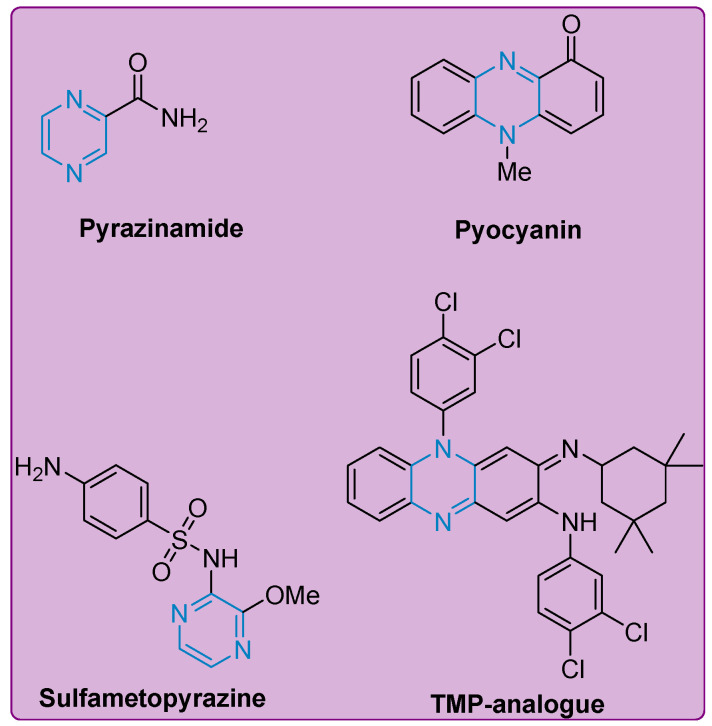
FDA-approved pyrazine-containing antibacterial agents.

Heterocycles are a crucial class of compounds in chemistry because of their numerous medicinal applications, importance in developing novel materials, and rampant presence in natural products. Numerous biological studies have investigated pyrazine- and phenazine-containing substances for their therapeutic relevance to human health and disease. In 2019, the WHO Model List of Essential Medicines included four pyrazine-based compounds (amiloride, pyrazinamide, bortezomib, and paritaprevir), the FDA-approved pyrazine-containing antibacterial agents are mentioned in Figure 1 [13,14].

Piddock and co-workers reported that heterocyclic nitrogen-containing compounds tested in *Salmonella Typhimurium* with an overexpressed AcrABToIC efflux pump showed hyper-susceptibility to antibiotics when acrAB was inactivated, reducing MIC values 2–4-fold in the presence of trimethoprim. Theobromine and norepinephrine also demonstrated synergistic effects with ciprofloxacin in these strains [15]. Foks et al. reported the tuberculostatic activity of the ammonium salts of pyrazines and found spiro-4-methyl-piperazin-1,1-1H-pyrazolo [3,4-b]pyrazin-3-ylamine chloride to be a potent compound [16]. Moreover, Gaikwad et al. found that pyrazine-containing thiazolines and thiazolidinediones are potential nontoxic anti-microbial agents against Gram-positive and Gram-negative bacteria, including *S. Typhi* [17]. Moreover, Ramachandran first described typhoid hepatitis and its histopathological features in 1974. Myopathy associated with typhoid fever has been increasingly reported in recent years. Additionally, elevated alkaline phosphatase levels are commonly observed in typhoid fever [18,19,20,21]. DNA gyrase is a highly attractive drug target, leading to the synthesis and characterization of numerous inhibitors. As a validated target for antimicrobial drug development, there is significant ongoing interest in creating novel gyrase inhibitors [22,23].

Alkaline phosphatase is a substrate-independent enzyme primarily responsible for AMP hydrolysis (although it also hydrolyzes other nucleotides). Ecto-5-nucleotidase, on the other hand, is a substrate-specific enzyme that solely hydrolyzes AMP to adenosine with the release of the phosphate group [24,25]. Compounds with nitrogen and amide linkages, such as pyrazole derivatives, have been demonstrated to inhibit human alkaline phosphatase activity [26]. An extensive literature review revealed that a number of compounds possessing Triazine, Pyridine, Pyrazol, and Pyrimidine have significant inhibitory potential towards alkaline phosphatase. In addition, in silico studies also provide a clue for the inhibitory potential of synthesized derivatives towards alkaline phosphatase [27].

In this work, we synthesized *N*-(4-bromo-3-methylphenyl)pyrazine-2-carboxamide and its derivatives (**5a**–**5d**) via a SMC (Suzuki cross-coupling) reaction by reacting the synthesized amide using several boronic acids. Further, we checked their activities against clinically isolated XDR *S.typhi* and enzymatic activities against the human alkaline phosphatase enzyme. To find the type of interactions, molecular docking studies were also carried out by using protein crystal structures (PDB ID: 1EW2) for enzymatic activities.

## 2. Results and Discussion

### 2.1. Chemistry

**Scheme 1:**
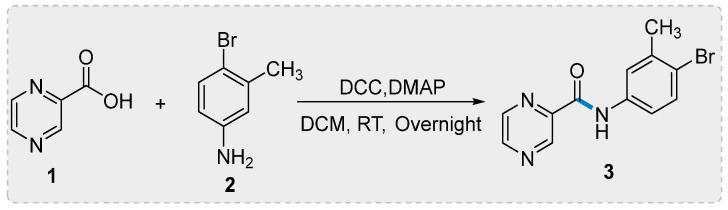
Synthesis of *N*-(4-bromo-3-methylphenyl)pyrazine-2-carboxamide.

**Scheme 2:**
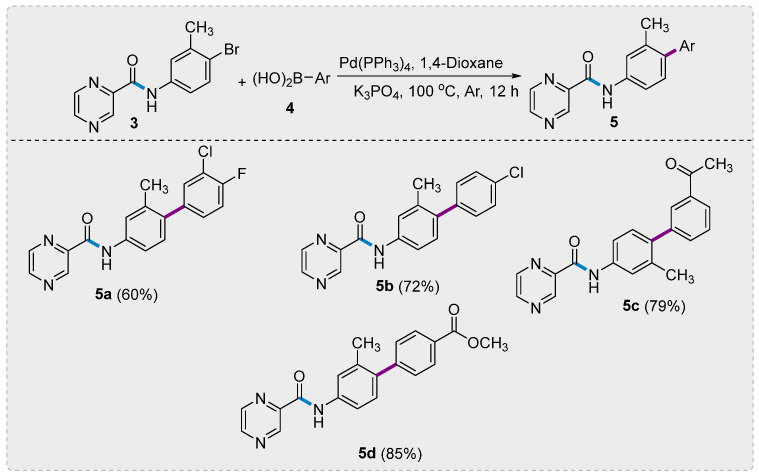
Arylation of *N*-(4-bromo-3-methylphenyl)pyrazine-2-carboxamide.

Using DCC/DMAP, pyrazine-2-carboxylic acid (**1**) and 4-bromo-3-methyl aniline (**2**) reacted to produce *N*-(4-bromo-3-methylphenyl)pyrazine-2-carboxamide, of which 83% was obtained (Scheme 1). *N*-(4-bromo-3-methylphenyl)pyrazine-2-carboxamide (**5a**–**5d**) arylated analogs were produced utilizing several aryl boronic acids and a palladium catalyst. A moderate to good yield (60–85%) of the derivatives of N-(4-bromo-3-methylphenyl)pyrazine-2-carboxamide (**5a**–**5d**) was afforded (Scheme 2).

### 2.2. Antibacterial Activity of the Compound against XDR-S. Typhi Pathogens

#### 2.2.1. Isolate Confirmation

The isolates were confirmed as XDR-*S. Typhi* after use of the Vitek 2 compact system, phenotypic confirmation, and MLST data analysis.

#### 2.2.2. Antibiogram of the Isolates

The MIC (µg/mL) for these pathogens was measured using the Vitek 2 compact system (BioMerieux, France) with various antibiotics. The antimicrobials tested were ampicillin, ceftriaxone, imipenem, meropenem, ciprofloxacin, cotrimoxazole, and azithromycin. The susceptibility was interpreted as per CLSI guidelines 2020. *S. Typhi* displayed resistance to the AWaRe (Access, Watch, and Reserve) WHO classes of antibiotics (Table 1).

#### 2.2.3. Antibacterial Activity of Compound against XDR-*S. Typhi*

**Figure 2:**
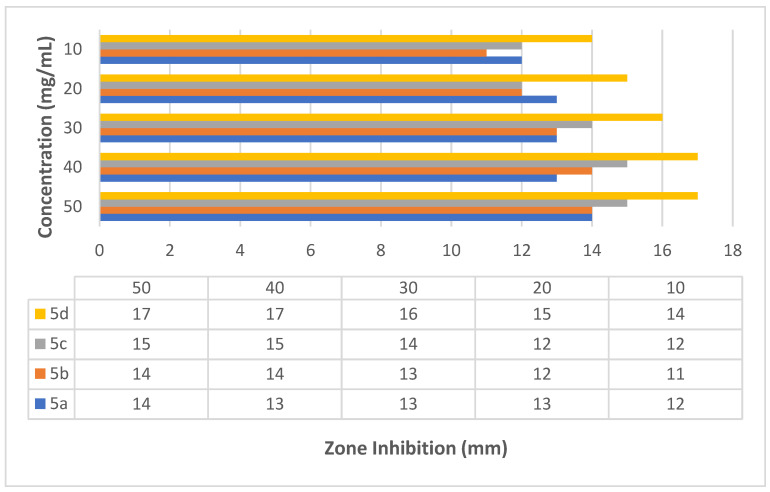
Agar well diffusion of different compounds (**5a**–**5d**) against XDR-*S. Typhi*.

Agar well diffusion was used to test the compounds (**5a**–**5d**) for antibacterial effects against XDR-*S. Typhi* at five doses (10, 20, 30, 40, and 50 mg/well). When the concentration of chemicals rises, so does the zone of inhibition (mm). When compound (**5d**) was concentrated at 50 mg/mL, it exhibited the largest zone of inhibition, measuring 17 mm, compared to the other compounds. However, compounds (**5a** and **5b**) showed (14 mm), and **5c** (15 mm) (Figure 2).

#### 2.2.4. MIC and MBC of the Compounds (**5a**–**5d**) against XDR-*S. Typhi*

**Figure 3:**
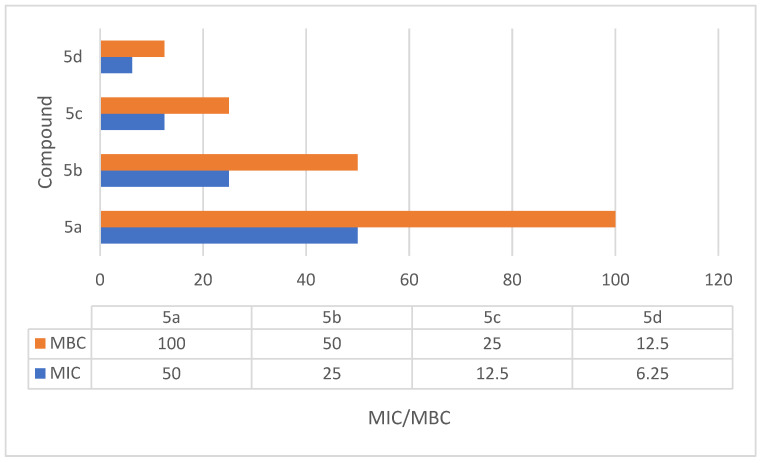
MIC and MBC (mg/mL) of compounds (**5a**–**5d**) against *XDR-S. Typhi.*

The results displayed the compound (**5a**) showed MIC 50 mg/mL and MBC 100 mg/mL, compound (**5b**) MIC 25 mg/mL and MBC 50 mg/mL, compound (**5c**) showed MIC 12.5 mg/mL and MBC 25 mg/mL, and compound (**5d**) showed MIC 6.25 mg/mL and MBC 12.5 mg/mL. The results showed that **5d** is the most potent compound while **5a** showed the least activity in the series as shown in Figure 3.

### 2.3. In Vitro Biological Activity

#### Enzyme Kinetics Studies

**Figure 4:**
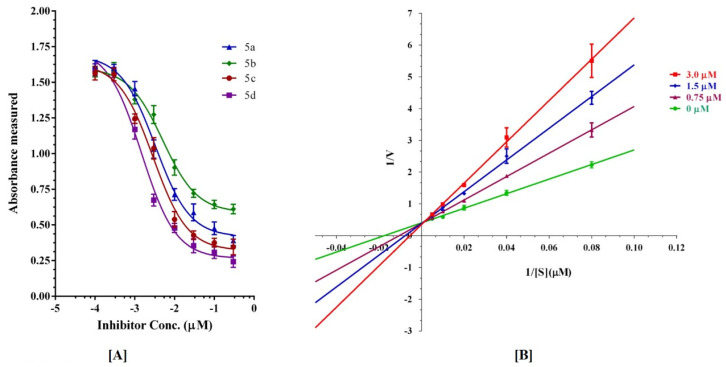
(**A**) IC_50_ curves for the synthesized compounds against alkaline phosphatase, (**B**) the Lineweaver Burk plot for enzyme kinetics studies showing that **5d** is a competitive alkaline phosphatase inhibitor.

Among the synthesized derivatives, **5d** was found to be the most potent inhibitor of alkaline phosphatase; therefore, this compound was subjected to enzyme kinetic studies using the Lineweaver Burk plot. The IC_50_ ± SEM for this compound was 1.469 ± 0.02 µM. Thus, four concentrations (0 µM, 0.75 µM, 1.5 µM, and 3 µM) of inhibitor were used against five different concentrations of substrate, including 0 µM, 12.5 µM, 25 µM, 50 µM, and 100 µM (Table 2 and Figure 4).

The specific pattern of the Lineweaver Burk plot exhibited that **5d** is a competitive inhibitor of alkaline phosphatase, which ensures the drugability of this synthesized derivative.

### 2.4. Docking Studies

#### 2.4.1. Protein–Ligand Interactions of the Synthesized Compounds (**5a**–**5d**) with the Target Protein (PDB ID: 5ztj)

Subunit DNA gyrase, a single chain of 312 amino acid residues, makes up a protein’s crystal structure. The resolution of this crystal structure is 2.4 Å. Since DNA gyrase is a vital enzyme necessary for *S. Typhi* cell proliferation, it is a prime candidate for treating bacterial infections. As a reference medication that selectively inhibits DNA gyrase, ciprofloxacin is utilized to compare the inhibitory potential of synthetic drugs towards this particular enzyme. Using PDB ID: 5ztj, the ciprofloxacin was docked in the target protein’s predicted active pocket during molecular docking studies. A literature review has shown that the binding energy of ciprofloxacin with the DNA gyrase of various bacterial strains varies from −4.0 to −10.0 kCal/mole [28,29,30,31]. In current study, the target protein’s active pocket showed a strong binding affinity with ΔG = −5.61799 kCal/mole. Ciprofloxacin established three hydrogen bonds with Lys550, Arg612, and Gly613. These hydrogen bond counts confer high binding interactions within the active pocket of the target protein. In addition to this, the amino acid residues of active pockets, including Tyr557, Asp576, and Arg615, interact with ciprofloxacin molecules through conventional carbon–hydrogen bonds. For this reference, the compound alkyl and π–alkyl interactions with Ile578 and Arg615 were also observed, as presented in Table 3.

**Figure 5:**
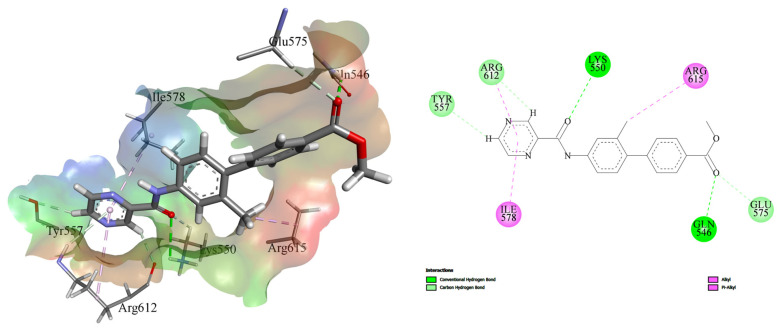
Putative binding mode of **5d**, within the active pocket of the target protein with PDB ID: 5ztj.

The binding energy for compound **5d** within the predicted active pocket of the target protein was found to be ΔG = −6.3891 kCal/mole, which is higher than that of the reference compound ciprofloxacin. Here, the molecule established two hydrogen bonds with Lys550 and Gln546, involving the oxygen of carbonyl carbons of **5d**. This molecule also established four conventional carbon–hydrogen bonds with Tyr557, Glu575, and Arg612, with pyrazine moiety and carbonyl carbon in the four carboxylate groups. In addition, π–alkyl interactions were also observed with Ile578 and Arg615, as presented in Figure 5. The binding modes of compounds (**5a**,**5b**,**5c**) with active pocket are shown (Supplementary Information Figures S4–S6).

#### 2.4.2. Protein–Ligand Interactions of the Synthesized Compounds (**5a**–**5d**) with the Target Protein (PDB ID: 1EW2)

All synthesized derivatives were docked against human placental alkaline phosphatase to determine the ideal conformational position inside the target protein’s active region. Human placental ALP protein is a class hydrolase and comprises a single chain of proteins containing 539 amino acids. This chain has embedded magnesium and zinc ions as cofactors. All the docked positions were examined based on hydrogen/hydrophobic interactions and free energy values (Kcal/mole). All the produced compounds show solid binding affinities with the target protein, as demonstrated by the docking results (Table 4).

**Figure 6:**
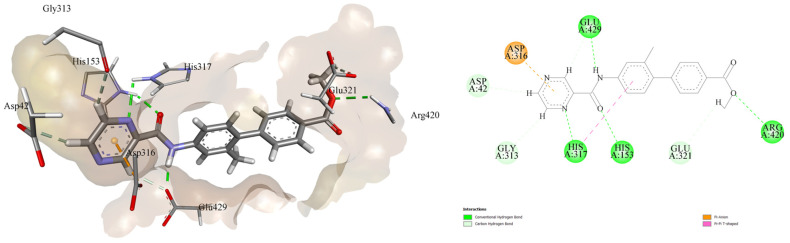
Putative binding mode of **5d** in the active pocket of the target protein with PDB ID: 1EW2.

**Figure 7:**
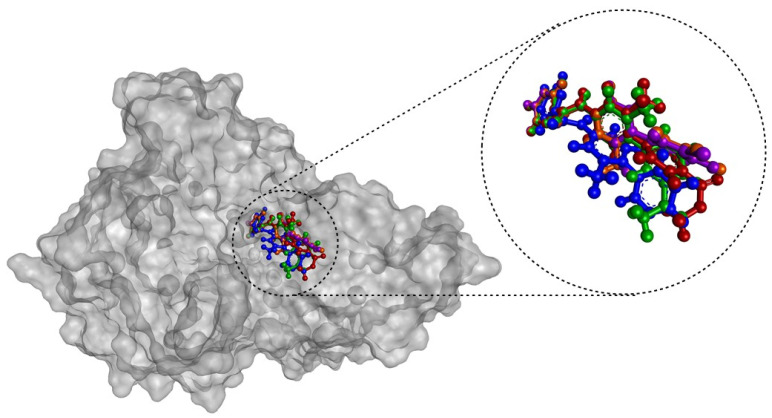
All the docked compounds were superimposed on the active pocket of the alkaline phosphate protein to check the RMSD of binding compounds and the target site.

Molecular docking studies of **5d** revealed that four hydrogen bonds were established involving His153, His317, Arg420, and Glu429 with the oxygen and nitrogen atoms of **5d**. In vitro studies have proved that this compound has the highest inhibitory potential for ALP, and molecular docking studies have further proved this highest activity among the synthesized compounds under this study in terms of the highest binding energy (ΔG = −7.5648 kCal/mole). The importance of these amino acid residue interactions has been well documented in previous studies [32,33]. Notably, this compound also formed three carbon–hydrogen interactions with Asp42, Gly313, and Glu321. The amino acid residues His317 and Asp316 were also found to establish hydrophobic interactions with the compound, as presented in Figure 6. The binding modes of compounds (**5a**,**5b**,**5c**) with active pocket are shown (Supplementary Information Figures S7–S9). All the docked compounds were superimposed on the active pocket of the alkaline phosphate protein to check the RMSD of the binding compounds and target site (Figure 7).

### 2.5. Pharmacokinetic Profile and ADME Evaluation

**Figure 8:**
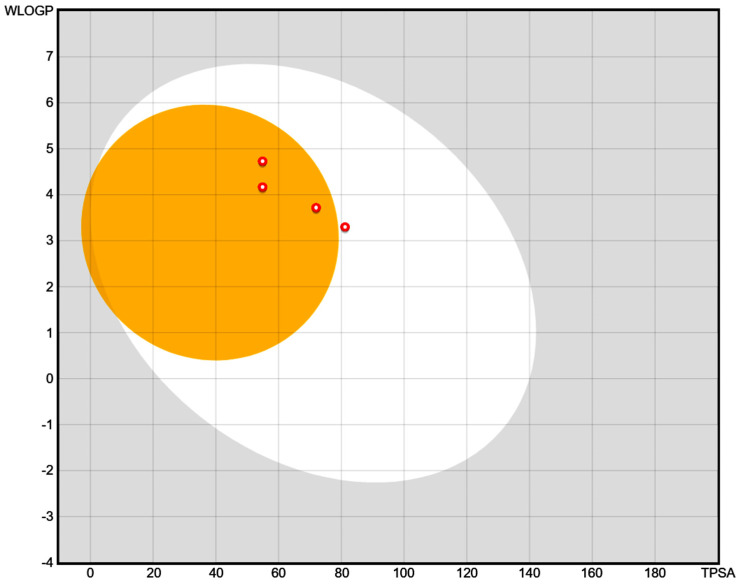
Boiled-egg plot of *N*-(4-bromo-3-methylphenyl)pyrazine-2-carboxamide derivatives for ADME evaluation.

The absorption, distribution, metabolism, and excretion of the synthesized derivatives were determined using an online in silico method (SwissADME) based on various algorithms to predict the ADME parameters. The important parameters are presented in Table 5, and a boiled-egg plot was generated, where the egg white portion indicates high gastrointestinal absorption and the egg yolk represents compounds that can cross the blood–brain barrier (BBB), as presented in Figure 8. All the synthesized compounds were predicted to exhibit high gastrointestinal absorption and have the ability to cross the blood–brain barrier, except **5d**, which was not able to cross the BBB. This in silico evaluation further indicated that all the synthesized compounds satisfied the Lipinski rules of five. The drugability of these compounds was also confirmed by applying a pan-assay interference compound (PAINS) filter, and none of the compounds exhibited significant similarities with the known PAINS. Thus, these synthesized compounds can be further evaluated and may be potential drug candidates [34].

### 2.6. Structure–Activity Relationship

**Figure 9:**
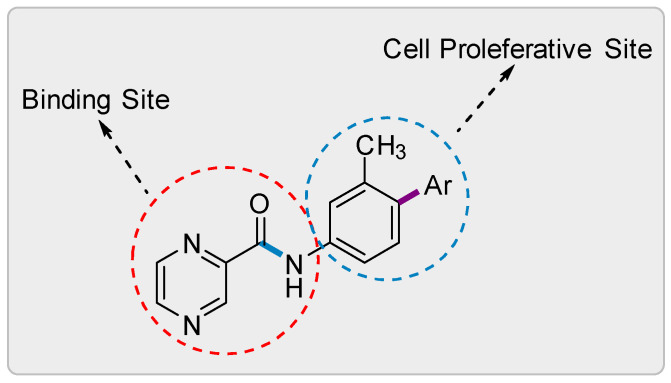
Structure–activity relationship of the main structural core of the synthesized molecules.

The docking studies indicated that the pyrazine heterocycle and amide group (highlighted in red) are involved in binding to the target site, whereas the aryl group (highlighted in blue) is associated with cell proliferation (Figure 9). Substituents on the aryl group enhance the activity; for instance, in compounds **5d** and **5c**, the ester group (CO_2_Me) and carbonyl group (COMe) form additional hydrogen bonds, which likely contribute to the increased activity. However, this enhancement is more pronounced when compared to other substituents, such as fluorine (F) and chlorine (Cl) groups.

## 3. Materials and Methods

### 3.1. General Information

All chemicals utilized in this research were obtained from Alfa Aesar and Sigma-Aldrich (USA). The SMC reactions were performed under an argon atmosphere. A rotary evaporator was employed to dry the synthesized molecules or reaction mixtures. The progress of the reactions was monitored using thin-layer chromatography (TLC) with silica gel 60 PF254 plates. The desired products were purified by flash column chromatography using silica gel with a mesh size of 230–400. Spectroscopic techniques, such as NMR (Bruker 400 MHz), were used to confirm the synthesized molecules in the presence of deuterated chloroform (CDCl_3_).

### 3.2. General Procedure for the Synthesis of N-(4-Bromo-3-methylphenyl)pyrazine-2-carboxamide

A 100 mL Schlenk flask was washed, oven-dried, and equipped with a magnetic stirrer bar. Pyrazine carboxylic acid (1.0 eq, 10 mmol), 50 mL DCM (Dichloromethane), 4-Bromo-3-methyl aniline (1.0 eq, 10 mmol), and DMAP (4-Dimethylaminopyridine) (0.2 eq, 20 mol%) were added to the flask. The reaction mixture was cooled to 0 °C in an isotherm while stirring continuously. Once the mixture reached 0 °C, DCC (N,N′-Dicyclohexylcarbodiimide) (1.1 eq, 11 mmol) was added, followed by an inert atmosphere. The isotherm was then removed. The reaction mixture was stirred at room temperature for 18 h and monitored by thin-layer chromatography (TLC). Upon completion, the reaction mixture was worked up with sodium bicarbonate solution followed by brine solution, using a separatory funnel. The organic layer was collected, and the solvent was removed with a rotary evaporator. The product was purified using column chromatography (hexane: ethyl acetate, 5:1) and then analyzed using spectroscopic methods (IR and NMR) [35,36].

#### N-(4-Bromo-3-methylphenyl)pyrazine-2-carboxamide (3)

White solid (83%, 2.42 g); ^1^H NMR (400 MHz, CDCl_3_) δ 9.61 (s, 1H), 9.49 (s, 1H), 8.80 (d, *J* = 2.4 Hz, 1H), 8.60–8.52 (m, 1H), 7.68 (d, *J* = 2.3 Hz, 1H), 7.52 (d, *J* = 8.6 Hz, 1H), 7.46 (dd, *J* = 8.6, 2.5 Hz, 1H), 2.41 (s, 4H). ^13^C NMR (101 MHz, CDCl_3_) δ 160.6, 147.6, 144.6, 144, 142.3, 138.8, 136.4, 132.8, 121.9, 120.0, 118.7, 23.1. Elemental Anal. Calcd: C_12_H_10_BrN_3_O (292.14) C, 49.34; H, 3.45; N, 14.38; Observed: C, 49.31; H, 3.46; N, 14.42 (Supplementary Information Figures S10 and S11).

### 3.3. General Procedure for the Synthesis of N-(4-Bromo-3-methylphenyl)pyrazine-2-carboxamides by Suzuki Coupling

A dried Schlenk tube equipped with a stirrer was supplemented with *N*-(4-bromo-3-methylphenyl)pyrazine-2-carboxamide (1.0 equiv, 1 mmol), tetrakis(triphenylphosphine)-palladium (0.05 equiv., 5 mol%), K_3_PO_4_ (2.0 equiv., 2 mmol), and aryl boronic acid (1.0 equiv, 1 mmol). Then, 8.25 mL of a 10:1 mixture of 1,4-dioxane and water was added under an inert argon environment. The reaction mixture was heated to 90 °C for 24 h and monitored by TLC. After completion, the mixture was cooled to room temperature, and water and ethyl acetate were added. The organic layer was separated and dried over sodium sulfate. The product was then purified via a column chromatography [36,37].

#### 3.3.1. N-(3′-Chloro-4′-fluoro-2-methyl-[1,1′-biphenyl]-4-yl)pyrazine-2-carboxamide (**5a**)

White solid (60%, 0.205 g); ^1^H NMR (400 MHz, CDCl_3_) δ 9.67 (s, 1H), 9.51 (d, *J* = 10.9 Hz, 1H), 8.82 (s, 1H), 8.60 (d, *J* = 7.2 Hz, 1H), 7.67 (s, 1H), 7.62 (dd, *J* = 8.3, 1.9 Hz, 1H), 7.33 (dd, *J* = 11.7, 4.7 Hz, 1H), 7.22–7.10 (m, 1H), 2.28 (s, 1H). Elemental Anal. Calcd.: C_18_H_13_ClFN_3_O (341.77) C, 63.26; H, 3.83; N, 12.30; Observed: C, 63.27; H, 3.81; N, 12.33 (Supplementary Information Figure S12).

#### 3.3.2. N-(4′-Chloro-2-methyl-[1,1′-biphenyl]-4-yl)pyrazine-2-carboxamide (**5b**)

White solid (72%, 0.233 g); ^1^H NMR (400 MHz, CDCl_3_) δ 9.67 (s, 1H), 9.51 (s, 1H), 8.80 (s, 1H), 8.58 (s, 1H), 7.67 (d, 1H), 7.62 (dd, *J* = 8.3, 1.8 Hz, 1H), 7.40–7.32 (m, 1H), 7.28–7.17 (m, 1H), 2.28 (s, 1H). ^13^C NMR (101 MHz, CDCl_3_) δ 159.6, 146.5, 143.6, 143.3, 141.4, 138.6, 136.3, 135.5, 135.4, 131.8, 129.5, 129.4, 127.3, 120.5, 116.3, 19.6. Elemental Anal. Calcd.: C_18_H_14_ClN_3_O (323.78) C, 66.77; H, 4.36; N, 12.98; Observed: C, 66.75; H, 4.37; N, 13.01 (Supplementary Information Figures S13 and S14).

#### 3.3.3. N-(3′-Acetyl-2-methyl-[1,1′-biphenyl]-4-yl)pyrazine-2-carboxamide (**5c**)

White solid (79%, 0.261 g); ^1^H NMR (400 MHz, CDCl_3_) δ 9.69 (s, 1H), 9.51 (d, *J* = 1.1 Hz, 1H), 8.81 (d, *J* = 2.4 Hz, 1H), 8.65–8.56 (m, 1H), 7.93 (ddd, *J* = 7.5, 5.0, 1.8 Hz, 2H), 7.70 (d, *J* = 1.7 Hz, 1H), 7.65 (dd, *J* = 8.2, 2.1 Hz, 1H), 7.56–7.45 (m, 2H), 7.28–7.21 (m, 1H). ^13^C NMR (101 MHz, CDCl_3_) δ 198.1, 160.6, 147.5, 144.6, 144.4, 142.4, 141.7, 137.5, 137.1, 136.6, 136.4, 133.9, 130.5, 129.1, 128.4, 126.8, 121.5, 117.4, 26.7, 20.6. Elemental Anal. Calcd. C_20_H_17_N_3_O_2_ (331.38) C, 72.49; H, 5.17; N, 12.68; Observed: C, 72.47; H, 5.18; N, 12.70 (Supplementary Information Figures S15 and S16).

#### 3.3.4. Methyl 2′-Methyl-4′-(pyrazine-2-carboxamido)-[1,1′-biphenyl]-4-carboxylate (**5d**)

White solid (85%, 0.295 g); ^1^H NMR (400 MHz, CDCl_3_) δ 9.63 (d, *J* = 15.7 Hz, 1H), 9.49 (d, *J* = 12.5 Hz, 1H), 8.78 (d, *J* = 2.3 Hz, 1H), 8.60–8.54 (m, 1H), 7.63 (ddd, *J* = 10.3, 7.4, 2.2 Hz, 1H), 7.46 (dt, *J* = 8.6, 5.5 Hz, 1H), 7.27–7.20 (m, 1H), 7.01–6.88 (m, 1H), 3.83 (s, 1H), 2.29 (s, 1H). ^13^C NMR (101 MHz, CDCl_3_) δ 160.6, 158.5, 147.5, 144.7, 142.4, 138.3, 136.5, 135.9, 133.6, 130.6, 130.3, 121.5, 117.3, 113.5, 55.3, 20.7. Elemental Anal. Calcd. C_20_H_17_N_3_O_3_ (347.37) C, 69.15; H, 4.93; N, 12.10; Observed: C, 69.18; H, 4.94; N, 12.12 (Supplementary Information Figures S17 and S18).

### 3.4. Isolate Identification

An amount of 3 mL of blood was drawn and deposited in BACTEC/Alert blood culture vials. The vials were incubated at 37 °C in a BACTEC/Alert automated system (BD, Marcy-l’Étoile, France). Positive specimens were then subcultured with blood and MacConkey agar (Oxoid, Hampshire, UK). The isolates were initially identified based on colony morphology and biochemically validated using the automated Vitek 2 system.

### 3.5. Agar Well Diffusion Method

The compounds’ antibacterial activity was determined via an agar well diffusion experiment towards XDR-*S*. Typhi. In brief, a 0.5 McFarland bacterial suspension was seeded onto a Mueller Hinton Agar plate, and wells in the agar were created using sterile 6 mm cork borers. Following that, 100 μL of each DMSO-diluted drug (at doses of 50 mg, 40 mg, 30 mg, 20 mg, and 10 mg) was added to the wells, and the plates underwent incubation overnight at 37 °C. The zones of inhibition (mm) were determined with a Vernier caliper. The experiment was run in triplicate, with a meropenem (10 µg) disk as the antibiotic control [10].

### 3.6. Minimum Inhibitory Concertation of Different Compounds against XDR-S. Typhi

As previously described, a microbroth dilution experiment assessed the MIC (mg/mL) of different compounds [38]. In a 50 mL Falcon tube, two to three isolated colonies were mixed with 20 mL of double-strength LB media and cultivated for a whole night at 37 °C. After that, the bacterial culture was diluted to an optical density (OD) of 0.07 at 600 nm or a 0.5 McFarland standard. A 96-well microtiter plate (Thermo Fisher Scientific, Leicestershire, UK) was used, with each well containing 100 μL of the chemical dilution (0.76, 1.56, 3.12, 6.25, 12.5, 25, and 50 mg). Next, each well received 100 μL of the bacterial solution. The positive control wells held the bacterial suspension in LB medium, while the negative control wells held 100 μL of LB media. The microtiter plates were kept in a shaking incubator (MaxQ^TM^ Mini 4450, Thermo Fisher Scientific) at 3 g for an entire night at 37 °C. The MIC was calculated by contrasting each well with the positive and negative control wells. Every technique was carried out three times.

### 3.7. Minimum Bactericidal Concentration against XDR-S. Typhi

The lowest concentration that prevents bacterial growth on an agar plate is the minimum bactericidal concentration (MBC; mg/mL). Nutrient agar plates (Oxoid, Hampshire, UK) were inoculated using a 10 μL sample from wells in the microtiter plate that did not exhibit any visible growth, and the plates were then aerobically incubated for a full day at 37 °C. After checking the plates for cell viability, any found colonies were noted as either having bacterial growth or not. Every technique was carried out three times.

### 3.8. Alkaline Phosphatase Inhibition Assay

A spectrophotometric assay was used to measure the activity of calf intestinal alkaline phosphatase (CIAP), adhering to the protocol outlined by Saeed et al. [33]. The reaction mixture comprised 50 mM Tris-HCl buffer (pH 9.5), 5 mM MgCl₂, and 0.1 mM ZnCl_2_. An amount of 5 μL of CIAP (0.025 U/mL) was added to the chemical (0.1 mM in 1% DMSO, *v*/*v*) and incubated for 10 min. Ten microliters of the substrate (0.5 milligrams of para-nitrophenylphosphate disodium salt, or p-NPP) were added to start the reaction, which was then incubated for thirty minutes at 37 °C. The p-nitrophenolate that had been produced was measured for absorbance at 405 nm using a 96-well microplate reader (OPTIMAx, tunable). Every experiment was run three times. Alkaline phosphatase’s reference inhibitor was Levamisol.

### 3.9. Molecular Docking Studies

The molecular docking of synthesized derivatives was performed in the active site of the bacterial enzyme DNA gyrase. The crystal structure of the gyrase of *Salmonella typhi* was retrieved from the RCSB database (https://www.rcsb.org/ (accessed on 20 March 2024)) with PDB ID: **5ztj**. DNA gyrase and topoisomerase IV are essential bacterial enzymes and important targets for developing novel antibacterial drugs. Therefore, we selected the DNA gyrase protein to compare the activities of newly synthesized compounds. The reason for choosing the DNA gyrase protein with PDB ID: 5ztj was that it is the only crystal structure available for *Salmonella typhi* DNA gyrase with the highest resolution of 2.40 Å. This protein’s crystal structure does not possess a co-crystallized ligand. Ciprofloxacin is the most effective therapeutic agent for *Salmonella typhi* infection, and targets its DNA gyrase [30,39,40]. Therefore, we docked ciprofloxacin in the reported active pocket of the DNA gyrase protein with PDB ID: 5ztj and compared the activities of the synthesized compounds with this standard inhibitor [41,42,43,44]. In addition to it, the crystal structure of human placental alkaline phosphatase with PDB ID: **1EW2** was also retrieved from the above-mentioned source with a resolution of 1.82 Å. Ligand preparation was started by drawing the chemical structures of synthesized compounds in the molecule structure builder tool of the Chem3D. Energy minimization of these compounds was carried out in the MMFF94x force field. Subsequently, these structures were saved as a database for docking studies. On the other hand, the protein’ crystal structure of the targeted enzyme was accomplished using Auto-Dock 4.2 [45].

## 4. Conclusions

This study highlights the urgent need for new therapeutic strategies to combat XDR-*S. Typhi*, which poses a significant public health threat due to its resistance to last-resort antibiotics. We successfully synthesized a series of pyrazine carboxamides (**5a**–**5d**) via the Suzuki reaction and evaluated their antibacterial activity against XDR *S. Typhi* using the agar well diffusion method. Among the synthesized compounds, **5d** demonstrated the highest antibacterial potency with a MIC of 6.25 mg/mL and an inhibition zone of 17 mm. Furthermore, compound **5d** showed significant alkaline phosphatase inhibition with an IC_50_ value of 1.469 ± 0.02 μM. Molecular docking studies confirmed the strong binding affinity of compound **5d** to the DNA gyrase protein, with a binding energy of ΔG = −7.5648 kCal/mole, surpassing the reference drug ciprofloxacin. The comprehensive in vitro and silico analyses establish compound **5d** as a promising candidate for further development as an antibacterial agent against XDR *S. Typhi*.

## Figures and Tables

**Table 1:** MIC (µg/mL) of antibiotics against XDR pathogens.

Antibiotics	MIC (µg/mL) Break Points	*S. Typhi*
AMP	≤8–≥32	≥512
CRO	≤1–≥4	≥256
IMP	≤1–≥4	2
MEM	≤1–≥4	2
CIP	≤0.25–≥1	≥8
SXT	≤2/38–≥4/76	≥4/152
AZT	≤16–≥32	4

AMP: ampicillin; CRO: ceftriaxone; IMP: imipenem, MEM: meropenem; SXT: co-trimethoprim; AZT: azithromycin.

**Table 2:** In vitro inhibitory potential of synthesized compounds against human placental alkaline phosphatase.

Compound Name	IC_50_ ± SEM
**5a**	3.281 ± 0.23
**5b**	5.022 ± 0.20
**5c**	2.864 ± 0.12
**5d**	1.469 ± 0.02

N = 3, the experiment was performed in triplicate format, and SEM was calculated.

**Table 3:** Interactions and binding free energy of synthesized compounds (**5a**–**5d**) with target protein (PDB ID: 5ztj).

Compound Code	Interactions	Free Energy ΔG (kCal/mole)
**5a**	Hydrogen bonding: Lys550, Arg612 C-H bonds: Glu575 π–Alkyl interactions: Tyr548	−6.1165
**5b**	Hydrogen bonding: Arg612 C-H bonds: Val540, Arg612 π–Alkyl interactions: Val540, Ile578, Arg612, Leu804, Val839, Met796 π–Sulfur interactions: Met796	−6.2706
**5c**	Hydrogen bonding: Gln546, Lys550, Arg612 C-H bonds: Glu575 π–Alkyl interactions: Arg612, Arg615	−6.1208
**5d**	Hydrogen bonding: Gln546, Lys550 C-H bonds: Tyr557, Glu575, Arg612 π–Alkyl interactions: Ile578, Arg615	−6.3891
Ciprofloxacin	H bonding: Lys550, Arg612, Gly613 C-H interactions: Tyr557, Asp576, Arg615 Alkyl and π–alkyl interactions: Ile578, Arg615	−5.61799

**Table 4:** Interactions and binding free energy of synthesized compounds (**5a**–**5d**) with the target protein (PDB ID: 1EW2)

Compound Code	Interactions	Free Energy ΔG (kCal/mole)
**5a**	H-bonding: His153, His317, Glu429 C-H bonding: Gly313, Glu429 π–Anion interaction: Asp316 π–π interaction: His317	−6.3287
**5b**	H-bonding: His153, His317, Glu429 C-H bonding: Gly313, Glu429 π–Anion interaction: Asp316 π–π interaction: His317	−6.2019
**5c**	H-bonding: His153, His317, Glu429 C-H bonding: Gly313, Glu429 π–Anion interaction: Asp316 π–π interaction: His317	−6.5274
**5d**	H-bonding: His153, His317, Arg420, Glu429 C-H bonding: Asp42, Gly313, Glu321 π–Anion interaction: His317 π–π interaction: Asp316	−7.5648

**Table 5:** Pharmacokinetics studies based on in silico method (SwissADME).

Code	TPSA ^a^	Lipinski Violation	PAINS ^b^	MLOGP ^c^	NRB ^d^	HBD ^e^	GI Absorption ^f^	HBA ^g^	BBB ^h^
**5a**	54.88	0	0	2.79	4	1	High	4	Yes
**5b**	54.88	0	0	2.41	4	1	High	3	Yes
**5c**	71.95	0	0	1.47	5	1	High	4	Yes
**5d**	81.18	0	0	1.74	6	1	High	5	No

^a^ Topological polar surface area, ^b^ pan-assay interference, **^c^** logarithm of partition coefficient between *n*-octanol and water, ^d^ number of rotatable bonds, ^e^ hydrogen bond donor, ^f^ gastrointestinal absorption, ^g^ hydrogen bond acceptor, ^h^ blood–brain barrier.

## Data Availability

Data are contained within the article.

## Supplementary Materials

The following supporting information can be downloaded at: https://www.mdpi.com/article/10.3390/ph17091241/s1, Figures S1 and S2 show antibacterial activities, Figures S3–S8 show docking studies, and Figures S9–S18 show Spectroscopic data.
