# Supplementary material for: Facile Synthesis of N-(4-Bromo-3-methylphenyl)pyrazine-2-carboxamide Derivatives, Their Antibacterial Activities against Clinically Isolated XDR S. Typhi, Alkaline Phosphatase Inhibitor Activities, and Docking Studies
Source: Pharmaceuticals (Basel). 2024 Sep 20;17(9):1241. doi: 10.3390/ph17091241 (PMC11434897; doi:10.3390/ph17091241)
Supplement: Supplementary file 1 [file pharmaceuticals-17-01241-s001.zip › pharmaceuticals-3145022-supplementary.pdf]

Supplementary information

Facile Synthesis of *N*-(4-bromo-3-methylphenyl)pyrazine-2-carboxamide derivatives; its Anti-Bacterial Activities Against Clinically isolated XDR *S. Typhi*, Alkaline Phosphatase Inhibitors activities and docking studies

Abdul Hannan Khan[1], Muhammad Bilal[1], Abid Mahmood[2], Nasir Rasool[1,*], Muhammad Usman Qamar[3], Muhammad Imran[4], Sebastian Ionut TOMA[5], Oana ANDREESCU[5]

[1] Department of Chemistry, Government College University Faisalabad, 38000, Pakistan; hannankhan786173@gmail.com (A.H.K.); muhammadbilalgcuf@gmail.com (M.B.); nasirrasool@gcuf.edu.pk (N.R.)

[2] Department of Pharmaceutical Chemistry, Government College University Faisalabad, 38000, Pakistan; abid9550@yahoo.com (A.M.)

[3] Institute of Microbiology, Faculty of Life Sciences, Government College, University Faisalabad, Faisalabad 38000, Pakistan; musmanqamar@gcuf.edu.pk (M.U.Q.)

[4] Chemistry Department, Faculty of Science, King Khalid University, P.O. Box 9004, Abha 61413, Saudi Arabia; imranchemist@gmail.com (M.I.)

[5] Faculty of Medicine, Transilvania University of Brasov; sebitom2002@yahoo.com (S.I.T); andreescu_o@yahoo.com (O.A.)

Correspondence: Nasir Rasool; nasirrasool@gcuf.edu.pk (N.R.)

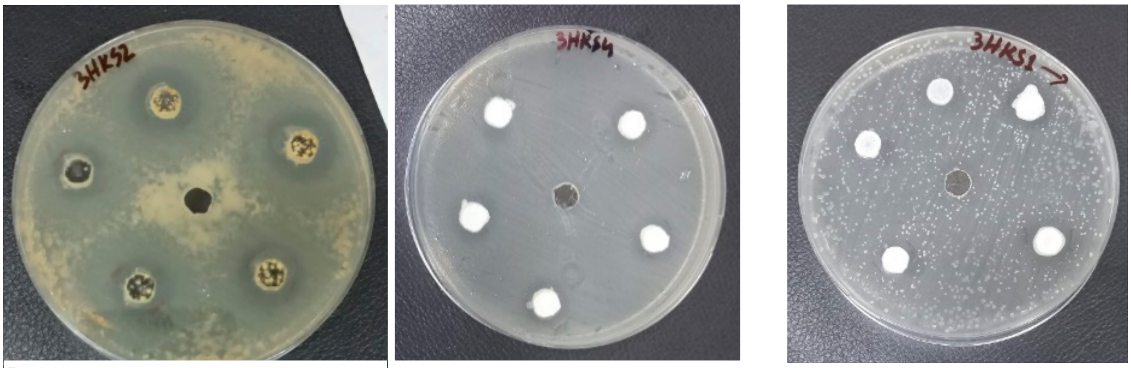

5a 5c 5d

Figure S1: Agar well diffusion of different compounds against *XDR S. Typhi*

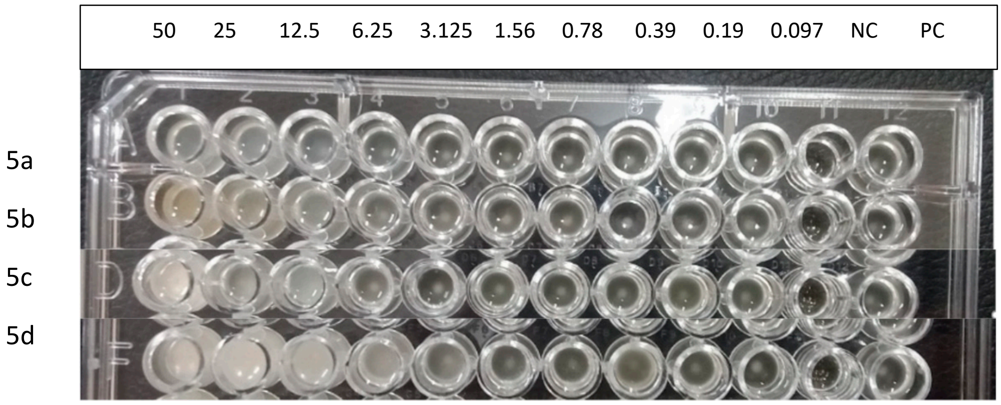

Figure S2: MIC of compounds against XDR S. Typhi. NC: negative control and PC: positive control

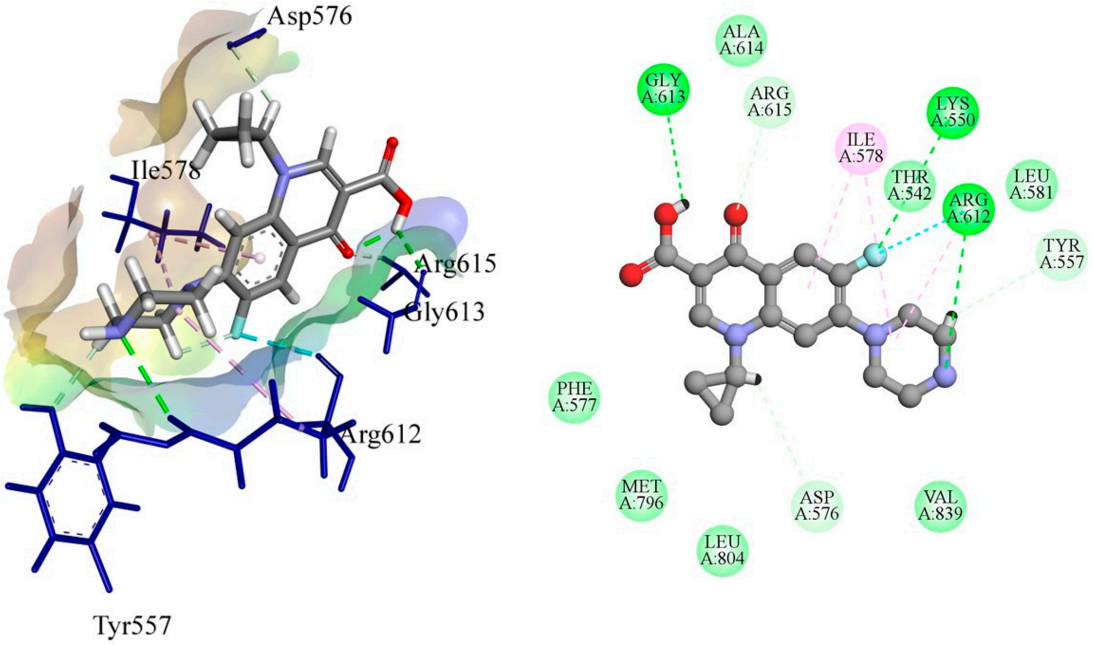

Figure S3: The putative binding mode of **Ciprofloxacin** within the active pocket of DNA gyrase protein PDB ID: **5ztj.**

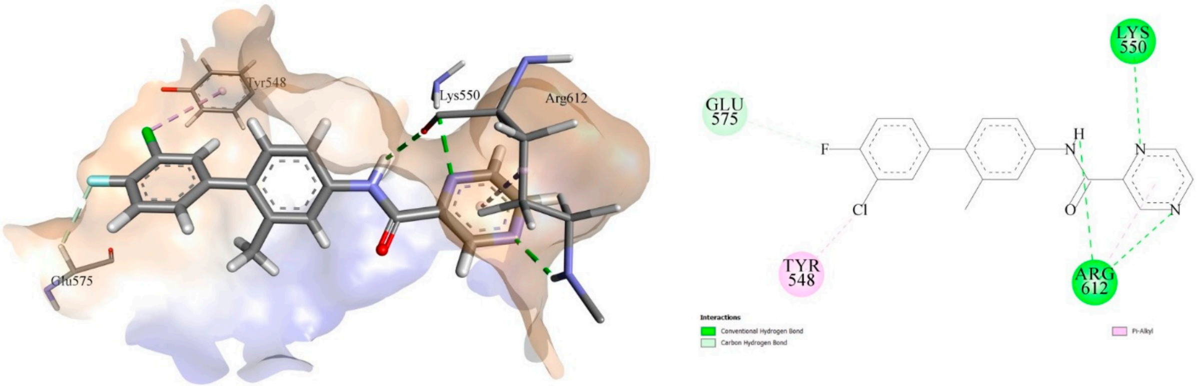

Figure S4. Putative binding mode of **5a**, within the active pocket of the target protein with pdb ID: 5ztj

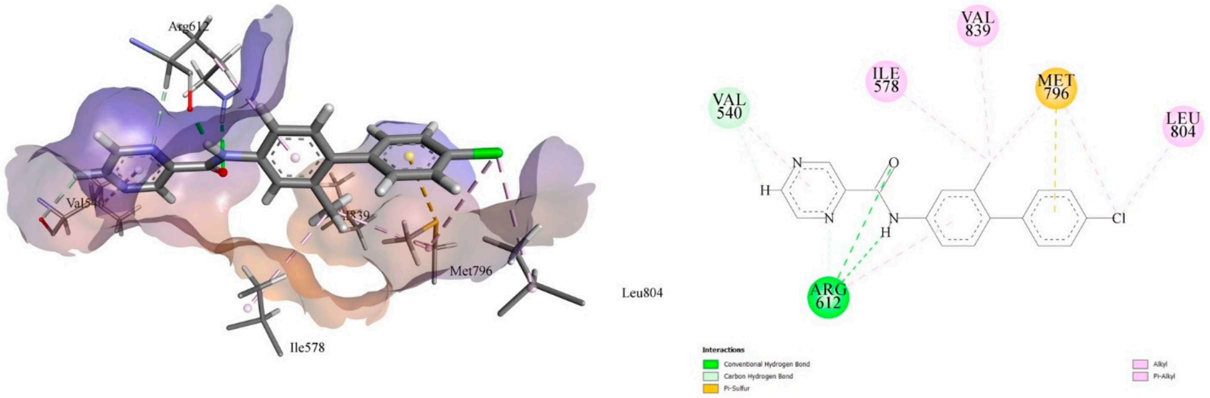

Figure S5. Putative binding mode of **5b**, within the active pocket of the target protein with pdb ID: 5ztj

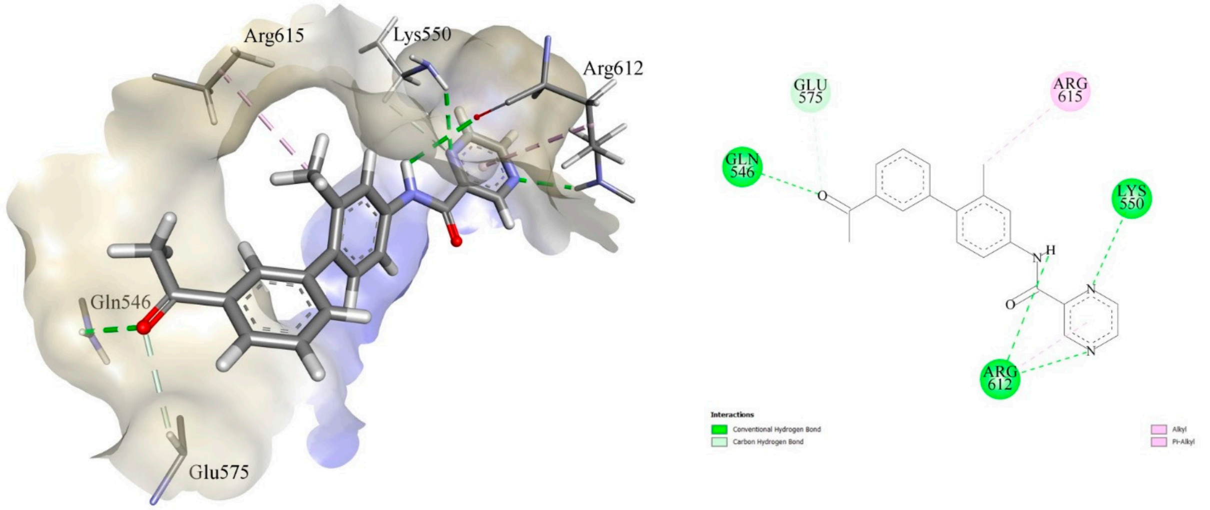

Figure S6. Putative binding mode of **5c**, within the active pocket of the target protein with pdb ID: 5ztj

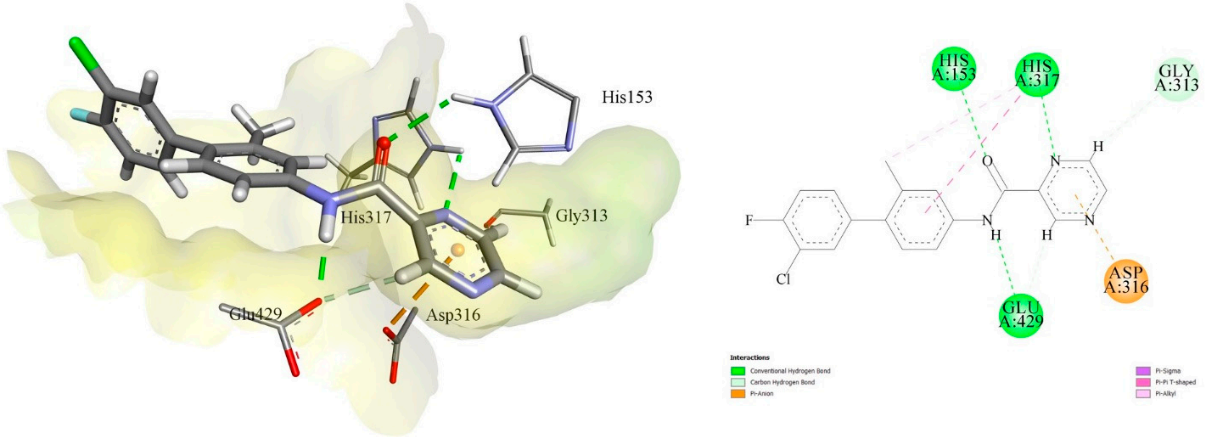

Figure S7: Putative binding mode of **$4HKS_2$** in the active pocket of the target protein with PDB ID: 1EW2 **(Supporting information)**

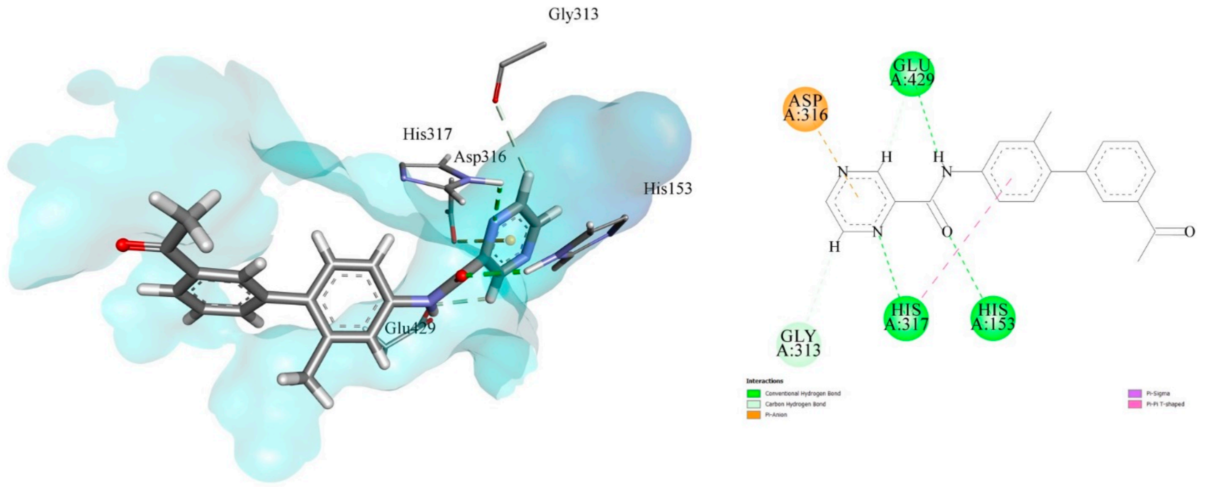

Figure S8: Putative binding mode of **$4HKS_4$** in the active pocket of the target protein with PDB ID: 1EW2 **(Supporting information)**

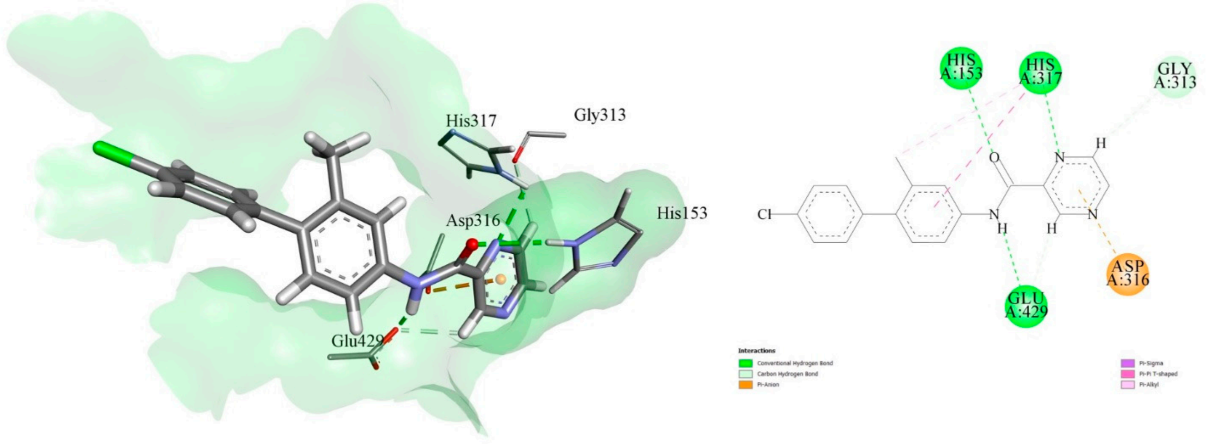

Figure S9: Putative binding mode of **5HKS$_7$** in the active pocket of the target protein with PDB ID: 1EW2 **(Supporting information)**

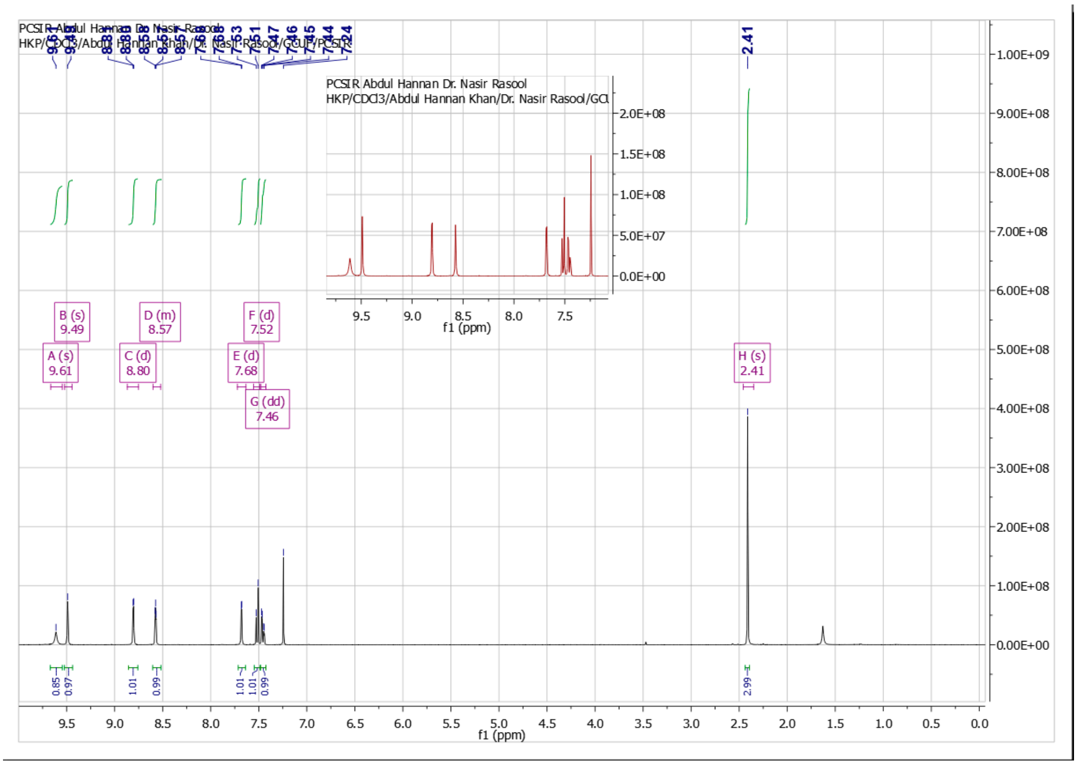

Figure S10: HNMR of N-(4-bromo-3-methylphenyl)pyrazine-2-carboxamide (3)

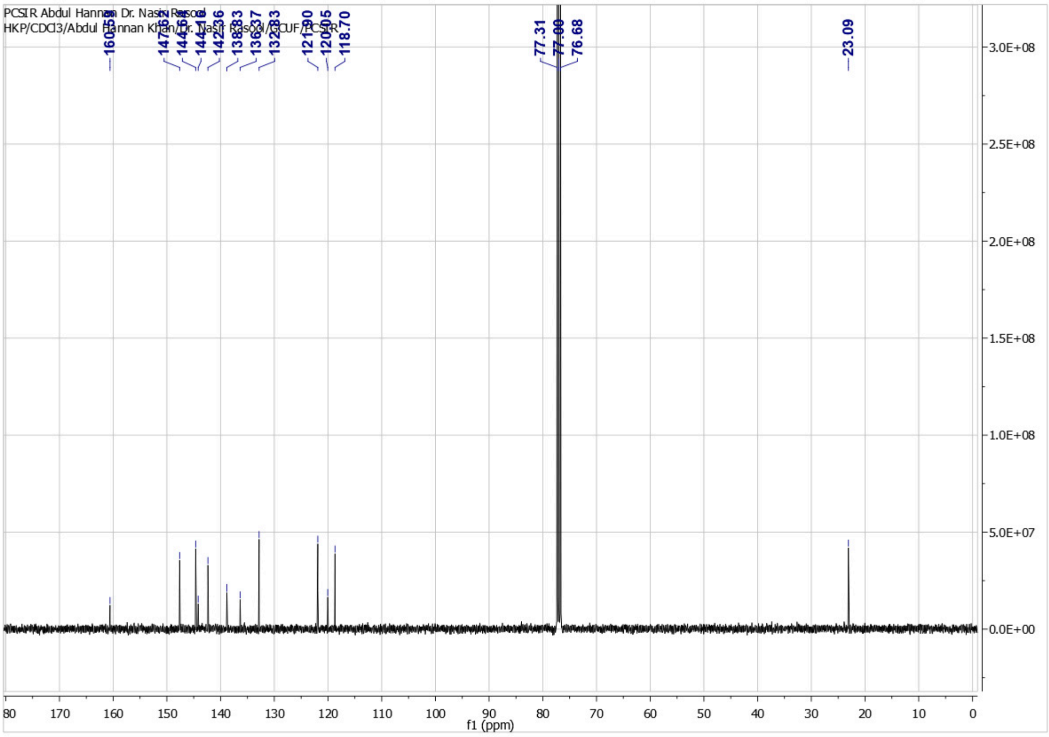

Figure S11: 13CNMR of N-(4-bromo-3-methylphenyl)pyrazine-2-carboxamide (3)

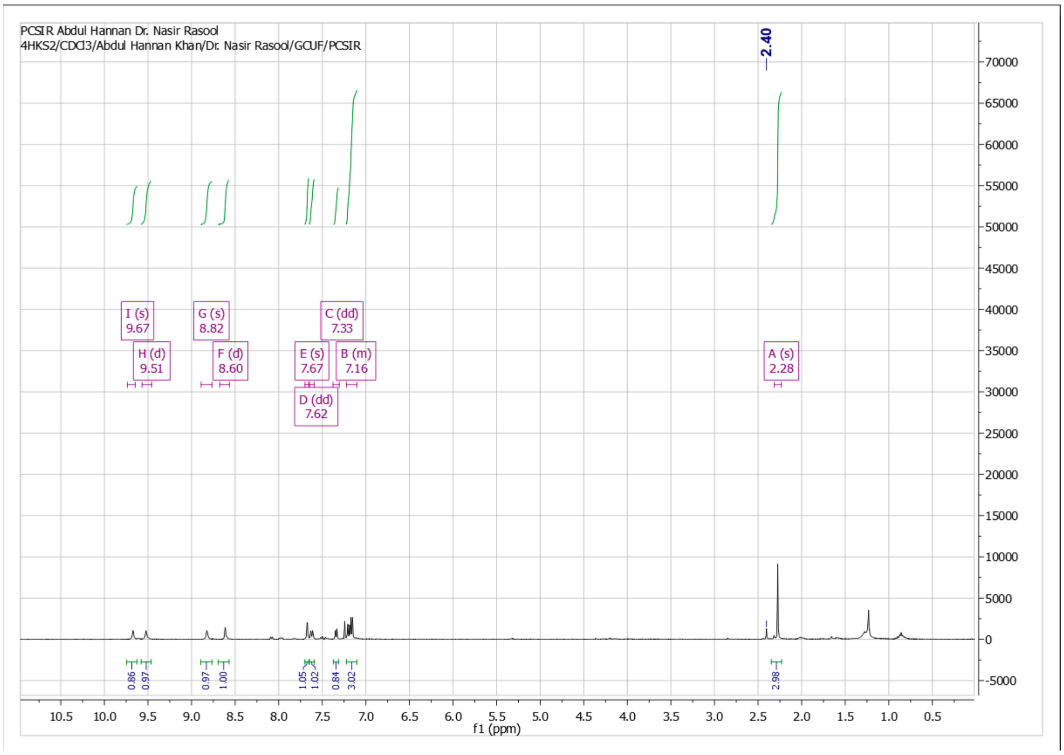

Figure S 12: N-(3'-chloro-4'-fluoro-2-methyl-[1,1'-biphenyl]-4-yl)pyrazine-2-carboxamide (5a)

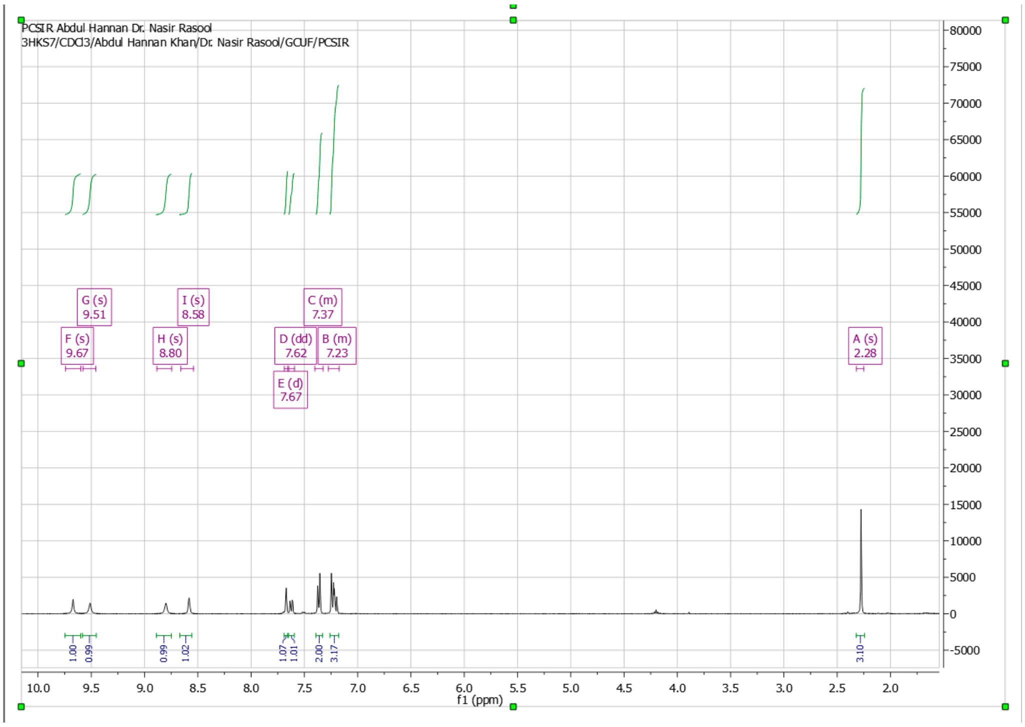

Figure S13: HNMR N-(4'-chloro-2-methyl-[1,1'-biphenyl]-4-yl)pyrazine-2-carboxamide (5b)

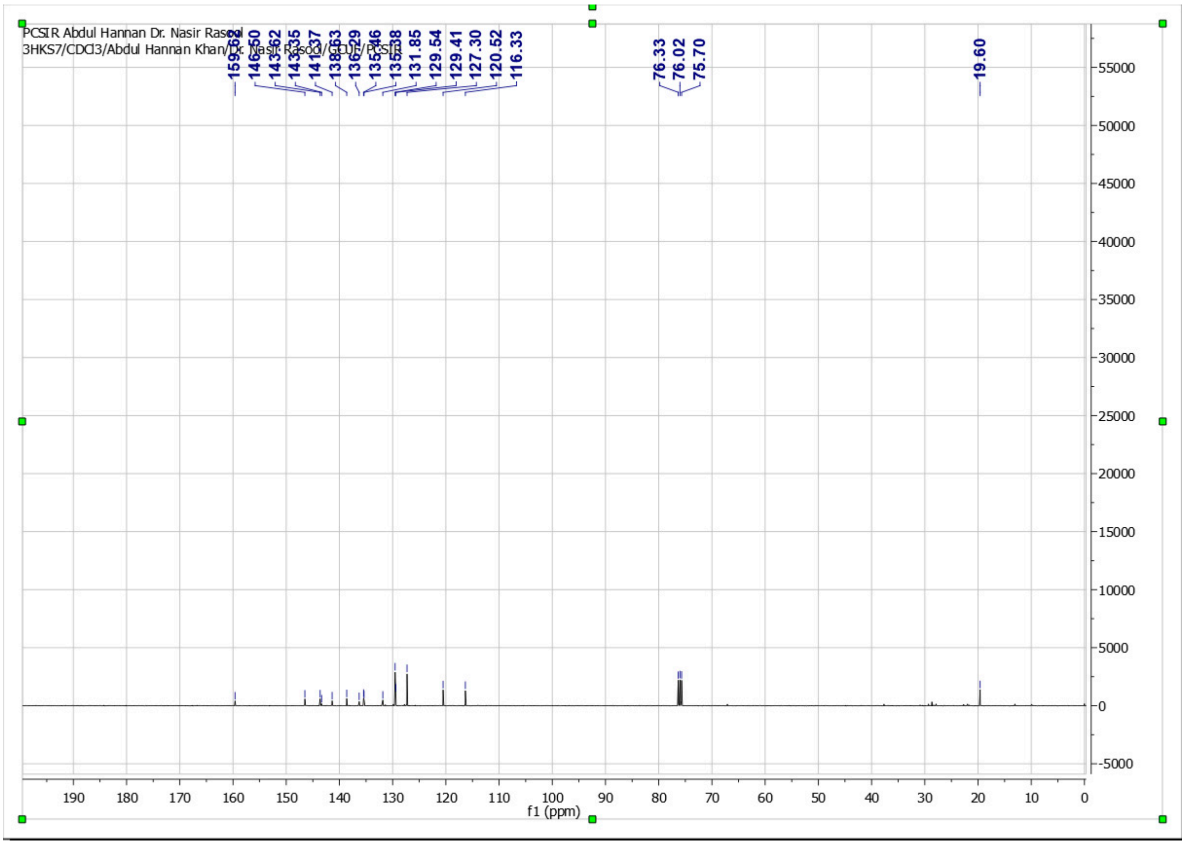

Figure S14: 13CNMR N-(4'-chloro-2-methyl-[1,1'-biphenyl]-4-yl)pyrazine-2-carboxamide (5b)

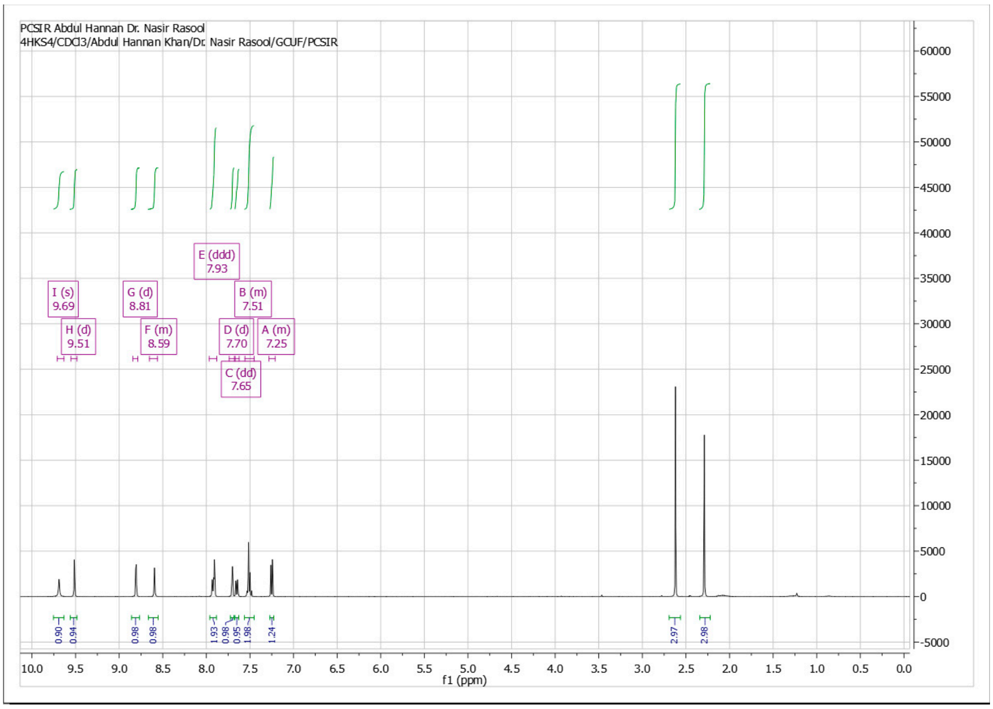

Figure S15: N-(3'-acetyl-2-methyl-[1,1'-biphenyl]-4-yl)pyrazine-2-carboxamide (5c)

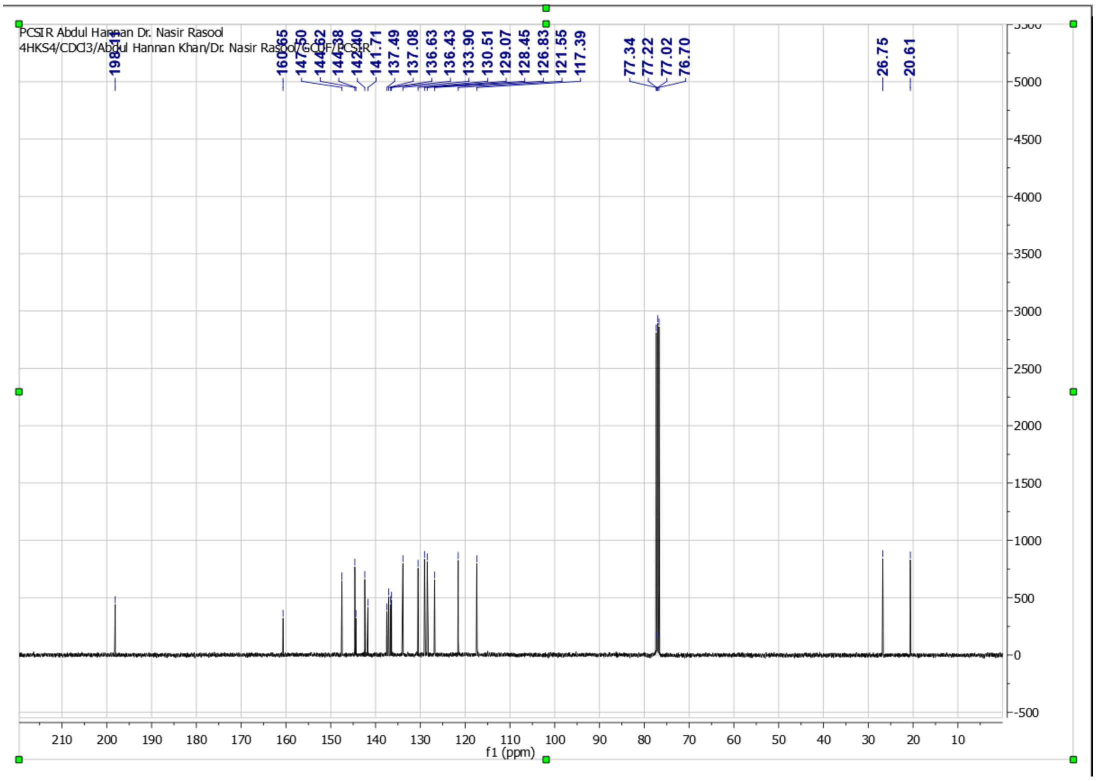

Figure S16: N-(3'-acetyl-2-methyl-[1,1'-biphenyl]-4-yl)pyrazine-2-carboxamide (5c)

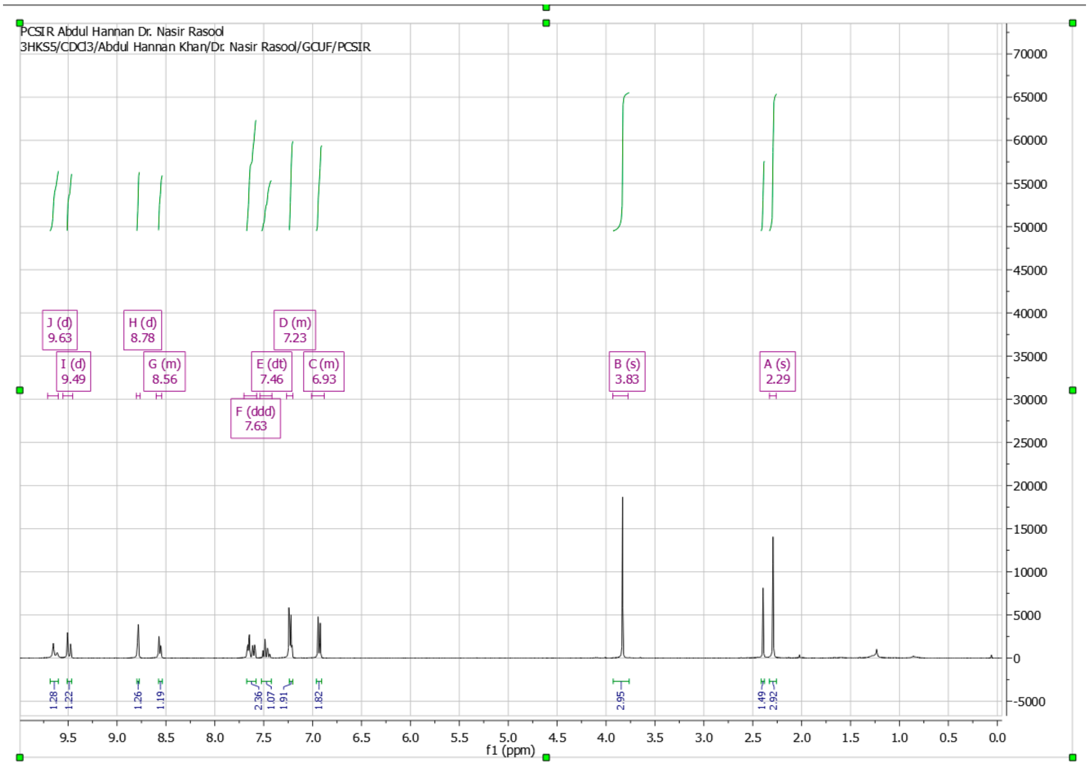

Figure S17: HNMR Methyl 2'-methyl-4'-(pyrazine-2-carboxamido)-[1,1'-biphenyl]-4-carboxylate (5d)

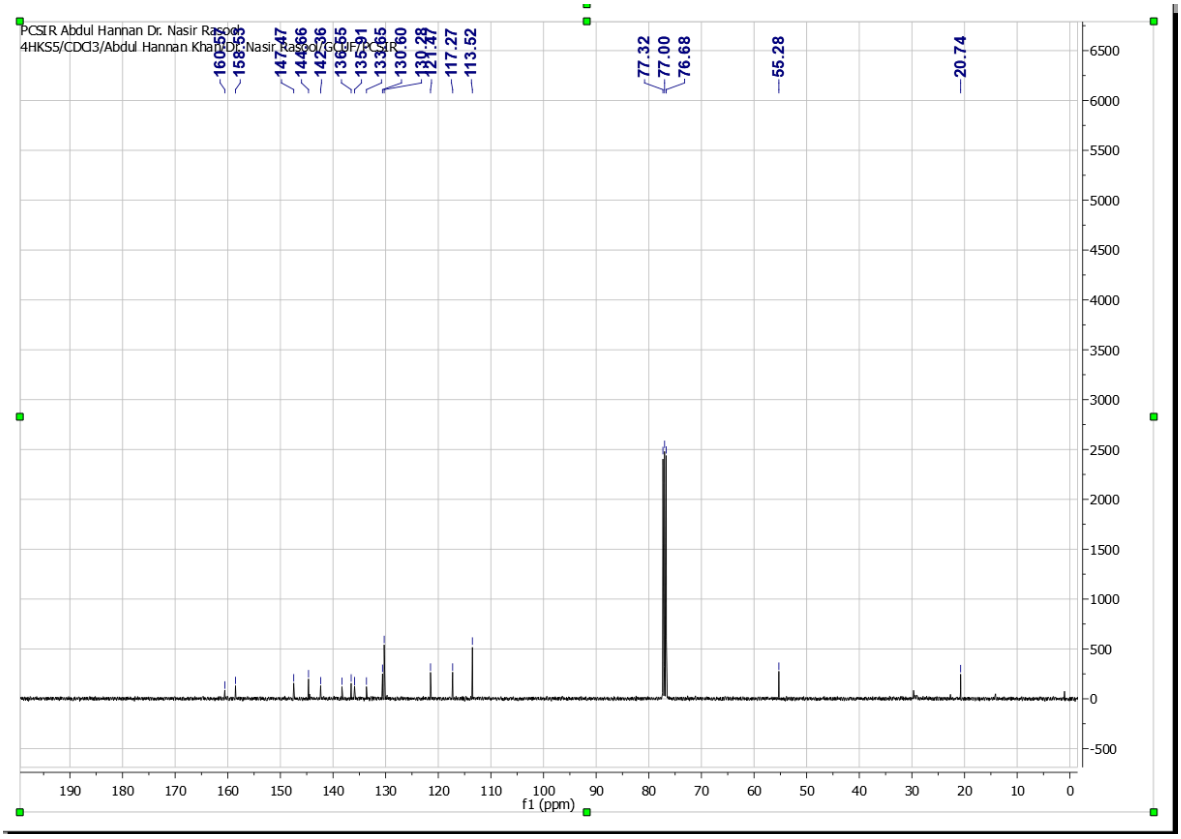

Figure S18: 13CNMR Methyl 2'-methyl-4'-(pyrazine-2-carboxamido)-[1,1'-biphenyl]-4-carboxylate (5d)
